# Climate-related experiences and harms in the wake of the COVID-19 pandemic: results from a survey of 152,088 Mexican youth

**DOI:** 10.1038/s41598-023-43305-5

**Published:** 2023-10-02

**Authors:** Ricardo Regules, Jessie Pinchoff, Ana C. Gomez-Ugarte, Tara F. Abularrage, Isabel Vieitez, Thoai D. Ngo

**Affiliations:** 1 Population Council-Mexico, Mexico City, Mexico; 2 Population Council, One Dag Hammarskjold Plaza #3, New York, NY 10017 USA; 3 Max Planck Institute for Demographic Research, Rostock, Germany

**Keywords:** Climate sciences, Environmental social sciences

## Abstract

The dual crises of COVID-19 and climate change are impacting the lives of adolescents and young people as they transition to adulthood in an uncertain world, yet they are often excluded from research and political discourse. We surveyed young people about their needs and experiences, critical to engaging them and designing effective programs and policies to address these intersecting harms. The 2022 round of a national online survey through the Violence Outcomes in COVID-19 Epoch (VoCes) Study surveyed 152,088 Mexican youth (15–24 years). Logistic regressions were implemented to identify characteristics associated with four climate responses (economic, work-related, receiving government support, or social network support). Overall, 8.1% of participants experienced a recent climate hazard, with major impacts including housing damage from floods, and crop/livestock losses from drought. Participants who experienced a climate hazard were more likely to have experienced a pandemic-related harm, suggesting a dual impact. Poor youth were more likely to report economic losses from both the pandemic and a climate event but least likely to receive government support. Economic effects from the pandemic are exacerbating climate-related harms, unequally threatening the poorest youth. Engaging young people in decision-making and supporting the most vulnerable youth is critical for the next generation to thrive.

## Introduction

The climate crisis is perhaps the most profound and imminent threat of the twenty-first century, already resulting in increasingly erratic weather patterns, rising temperatures, stronger storms, and persistent droughts^1^. The causes and impacts of climate change are unequal and exacerbate underlying social, economic, and political factors, resulting in disparities in health, economic security, and well-being^2^. Climate change threatens future generations, with recent research highlighting that children born in 2020 will experience a two to seven-fold increase in extreme events, particularly heatwaves, compared to those born in 1960^3^. There are both direct and indirect impacts on adolescents and young people, with both short- and long-term repercussions as they are exposed to recurring and cumulative risks throughout their lives^2,4,5^. This includes navigating the transition to adulthood during a time of systemic instability and change related to displacement, shifting livelihood opportunities, economic dislocation, and conflict that may result in worse health outcomes and well-being^2^. Despite being the most impacted, the world’s largest generation of 1.8 billion young people are often not engaged in research or policy related to the climate crisis. However, this is beginning to shift with youth advocates and growing youth movements bringing these and other diverse voices into the conversation^6,7^.

Mexico’s geography and underlying inequalities make certain communities vulnerable to extreme weather events that threaten transportation, power, water infrastructure, and agriculture^8,9^. In Mexico, climate models predict annual temperatures may increase by 3.7 °C by 2090^9,10^. An estimated 70% of Mexico’s territory is vulnerable to droughts that are predicted to reduce farming surface from 40 to 25%, impacting the country’s capacity to feed its growing population^11^. People living in rural areas who rely on agriculture and livestock for their livelihoods are particularly vulnerable as they are dependent on natural resources^12^. Recent studies show that exposure to climate shocks is connected with climate change risk perception and willingness to change behaviors^13,14^. One study conducted in Mexico found that younger people were significantly more willing to take on adaptation measures aimed at conserving water^14^.

Against this backdrop, the COVID-19 pandemic struck in 2020, bringing global inequalities into sharp relief, with the largest single year increase in global inequality and poverty since 1990^15^. In Mexico, the impact of the COVID-19 pandemic has been severe, with a high COVID-19 case fatality rate, widespread socioeconomic impact, and the world’s third-highest death toll^16,17^. According to the 2020 population census, in Mexico 25% of the population (31 million) are adolescents and young adults (15–29 years)^18^. Compared to pre-pandemic, youth are less likely to be enrolled in school, spend 25% less time on schoolwork, and also increased their employment and hours worked^19^. The spread of cases and fatalities associated with COVID-19 reflect existing patterns of spatial segregation and inequalities in Mexico, with marginalized municipalities with high population density, low-income neighborhoods, and individuals in economically unfavored sectors most impacted^16,20^. There is some geographic overlap between the pandemic and climate, but the strongest link is that the most economically and socially vulnerable are doubly harmed^21,22^. Individual adaptive capacities are constrained by structural conditions and socioeconomic inequalities related to access to material and financial resources^23^. Poorer individuals are generally more likely to be harmed by climate as they have fewer assets to help them recover from climate shocks and stresses, their livelihoods are more likely to depend on a climate sensitive sector (e.g. agriculture), and they are more likely to live in areas with higher exposure to climate extremes^24^.

Although it is clear that the climate crisis will significantly affect the lives of children and youth, there is limited data to date about how it may shape their lives, and how they will engage with climate-related challenges as societies emerge from the pandemic^2^. Studies from Mexico highlight the risks of climate change, but are not focused on young people^13,14,25^. The Violence Outcomes in COVID-19 Epoch (VoCes) Study is a longitudinal survey regarding the impacts of COVID-19 on adolescents and young adults (15–24 years) in Mexico, leveraging an innovative online platform to engage and reach youth across the country with an online survey^26^. This paper uses data from the 2022 wave of the VoCes survey to explore adolescent and young adults’ perceptions of how climate change is impacting their lives in the wake of the COVID-19 pandemic. Our results highlight how vulnerabilities and geographies can intersect, with implications for the development of more targeted and nuanced policy formulation and implementation.

## Methods

### Survey

A longitudinal study was conducted by Population Council Mexico, in collaboration with the National Institute of Youth (IMJUVE) and the National Center for Gender Equity and Reproductive Health (CNEGSR). VoCes was designed to examine the multi-dimensional impact of the COVID-19 pandemic and accompanying mitigation measures on the experience and perception of violence among adolescent and young adults (15–24 years of age) in Mexico. The first survey round was conducted between November 2020 and February 2021 and focused on the potential harms of COVID-19 lockdown policies. The second wave was conducted in 2022 to follow up, and a module of questions related to climate change was added, as this topic was identified as critical to the lives of adolescents and young people.

The questionnaire took approximately 35 minutes to complete, and asked a series of questions regarding demographics, experience with COVID-19 including infection, vaccination, and harms (dropping out of school, economic insecurity, and various social harms). The second round included an additional module regarding experiences of climate related hazards (drought, heatwaves, hurricanes and flooding), and various economic and social harms and adaptive responses related to these exposures.

### Sampling

During the pandemic, in-person data collection was not possible, so an online platform was developed. The target population for the survey was adolescents (15–17 years) and young adults (18–24 years) living in Mexico. Recruitment occurred via an open invitation through social media, radio and television spots, and a targeted invitation made by the IMJUVE, ministry of health, ministry of education, and different educational and indigenous peoples authorities^26^. Informed consent was obtained from all participants. In addition, a second consent was requested to provide their email or cell phone number for future rounds of the study. In Round 1 55,692 completed the survey, in Round 2 168,407 completed the survey (with overlap).

### Data analysis

Respondent’s characteristics were tabulated by reported experience of any climate related hazard. Maps at the state level were constructed to visualize geographic variation in exposure to flooding, hurricanes, drought or heat waves over the last 12 months. Respondent characteristics included gender (male, female, trans or non-binary), a 4-level variable consisting of working or in school (in school only, working only, study and working, neither studying nor working), a five-level region variable (central, south, central north, north west, or north), and a two-level age variable (15–17 vs 18–24 years).

Two variables were used to define exposure to a climate event, first a binary response to the question “in the last 12 months did you experience any climate related event?”, and then the follow up response to “which event impacted you the most” to which respondents could select flooding, hurricane, heat wave, or drought. Respondents were then asked how they were impacted (and could select more than one): damage to the home, lost work or could not work, harm to the family business, left school, damage to agriculture or livestock, severe health impacts, or not impacted. Possible adaptive responses or actions included economic responses (combined from to use savings or borrow money), work-related responses (combined from work more hours or search for technical training), receiving government support, or relying on family/friends and social network for support. 

Two pandemic related harms were explored. One question asked if anyone in the respondents’ household had died of COVID-19 in the past year (yes or no response options). Another asked if respondents had lost income due to the pandemic (very likely, somewhat likely, a little, probably not, don’t know). These responses were collapsed into a binary variable with yes for those who reported they had very likely lost income. Respondents were asked if from March 2021 to the present (about 1 year), had the respondent and their family been able to … “buy enough good for everyone in the house”, “pay important bills, for example rent”, or “buy the necessary medicines for some of the people at home”. Respondents could reply always, almost always, sometimes, almost never, or never. These responses were collapsed into binary variables, with yes for  those who reported always or almost always could afford. A variable was also generated for experiencing none vs one or more of these pandemic harms. 

Four logistic regression models were fit for each of the climate related response or actions: (1) economic response; (2) work related response; (3) receiving government support; and (4) receiving social network support. Each model explored how each type of climate hazard (flood, drought, hurricane, heat wave) related to the outcome, adjusting for age, gender, working or in school, wealth tercile, and region of residence. Additionally, the models explored if experiencing pandemic related household income loss was associated with the climate related response outcome.

### Ethical approval

The survey received institutional review board (IRB) approval from the Population Council IRB (IRB Research Protocol No. 949). All methods were performed in accordance with relevant guidelines and regulations.

## Results

A total of 152,088 (out of 168,407) participants completed the questions on climate, with an average age of 16.4 years and 58% identifying as women (n = 86,491 participants). Most participants (n = 112,038, 81%) were in school at the time of the survey, and about a quarter were studying and working for income (n = 24,375, 18%) (Table 1). Almost one in ten participants (n = 12,349, 8.1%) reported experiencing any climate related hazard in the last year. Respondents in the poorest wealth tercile were most likely to report exposure to a climate hazard (5714 respondents, 46%), compared to the middle or wealthiest tercile. There was geographic variation in exposure to a recent climate hazard with those in living in rural areas (n = 9260, 75%) and the Southern region (n = 3154, 26%) more likely to have been exposed.

**Table 1:** Demographic characteristics of respondents, by exposure to any climate event in the last 12 months.

	Not exposed to a climate event in last 12 months N = 139,739	Exposed to a climate event N = 12,349	Total N = 152,088	P value
Age category				< 0.001
15–17 years	122,198 (87%)	10,594 (86%)	132,792 (87%)	
18–24 years	17,541 (13%)	1755 (14%)	19,296 (13%)	
Gender				< 0.001
Men	54,912 (40%)	4886 (40%)	59,798 (40%)	
Women	79,581 (58%)	6910 (57%)	86,491 (58%)	
Trans/nonbinary	2745 (2%)	323 (3%)	3068 (2%)	
Wealth tertile				< 0.001
Poorest tertile	48,489 (35%)	5714 (46%)	54,203 (36%)	
Middle	67,517 (48%)	5237 (42%)	72,754 (48%)	
Wealthiest tertile	23,702 (17%)	1398 (11%)	25,100 (17%)	
Working or studying (in school)				< 0.001
Studying	103,789 (82%)	8249 (74%)	112,038 (81%)	
Working	553 (0%)	87 (1%)	640 (0%)	
Studying and working	21,670 (17%)	2705 (24%)	24,375 (18%)	
Not studying and not working	458 (0%)	48 (0%)	506 (0%)	
Region				< 0.001
South	15,229 (11%)	3154 (26%)	18,383 (12%)	
Central	98,668 (71%)	7041 (57%)	105,709 (70%)	
Central-North	6563 (5%)	490 (4%)	7053 (5%)	
North-West	2796 (2%)	324 (3%)	3120 (2%)	
North	16,483 (12%)	1340 (11%)	17,823 (12%)	
Urban	112,914 (81%)	9260 (75%)	122,174 (80%)	< 0.001
Rural	26,825 (19%)	3089 (25%)	29,914 (20%)	
Pandemic impacts				
Someone in my home died of covid	6503 (5%)	794 (7%)	7297 (5%)	< 0.001
At least 1 pandemic economic harm (1–3 vs 0)	29,938 (21%)	4496 (36%)	34,434 (23%)	< 0.001
Lost income due to pandemic	17,469 (13%)	2661 (22%)	20,130 (13%)	< 0.001

**Figure 1:**
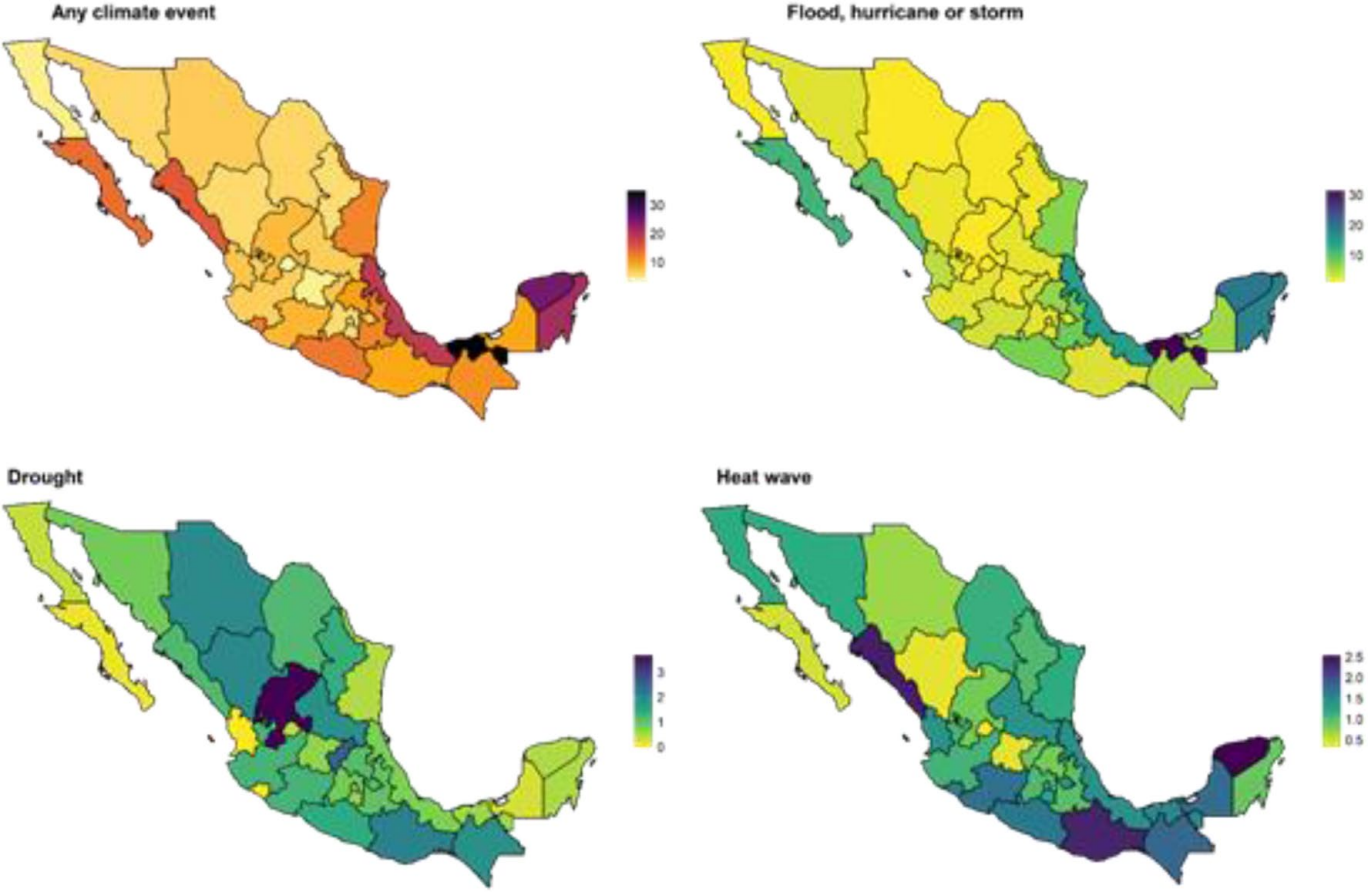
Percentage of youth who reported experiencing a climate event in the last 12 months preceding the survey, by type of climate event.

Among those who experienced any climate event in the last year, the most reported climate hazard was floods (n = 4209, 34%), followed by hurricanes (n = 2198, 17.8%), heat waves (n = 1857, 15.0%) and drought (n = 1547, 12.5%) (Table 2). Hurricanes and flooding were more frequently reported in coastal states of the Southern region, with heat waves and droughts more reported in inland parts of Mexico that rely on agriculture. Figure 1 highlights the geographic distributions for each type of hazard most reported per state. Among respondents who experienced a recent climate event, the most commonly reported harm was damage to the home, most frequently reported due to floods (n = 2989, 71%) or hurricanes (n = 1521, 69%). Harm to crops or livestock was reported by about half of respondents who experienced a drought (n = 862, 56%) and about a fifth of those who experienced heat waves (n = 392, 21%) (Table 2). Health impacts were most likely to be reported among those exposed to heatwaves, with almost a quarter of those who experienced heat waves reporting an impact on their health (n = 451, 24%). Those who experienced any climate related harm were also more likely to have experienced a pandemic-related harm, with 36% (n = 4496) of those who had also been impacted by a climate event reporting someone in their household had died and 22% (n = 2661) reporting they had very likely lost income due to the pandemic.

**Table 2:** Impacts and actions taken for each type of climate hazard reported among respondents who experienced any climate hazard in the last 12 months.

Exposed to…	Flood N = 4209	Drought N = 1547	Hurricane N = 2198	Heat N = 1857	P-value
Climate related harms					
Damaged the home	2989 (71%)	268 (17%)	1521 (69%)	314 (17%)	< 0.001
Lost work or couldn’t work	303 (7%)	126 (8%)	188 (9%)	108 (6%)	< 0.001
Dropped out of school	149 (4%)	26 (2%)	93 (4%)	45 (2%)	< 0.001
Damage to crops/livestock	331 (8%)	862 (56%)	300 (14%)	392 (21%)	< 0.001
Health impacted	105 (3%)	68 (4%)	69 (3%)	451 (24%)	< 0.001
Didn’t affect me	636 (15%)	190 (12%)	271 (12%)	610 (33%)	< 0.001
Pandemic related harms					
Someone in my home died of COVID	257 (6%)	121 (8%)	99 (5%)	145 (8%)	< 0.001
One or more economic harm (0 vs 1+)	1484 (35%)	613 (40%)	716 (33%)	778 (42%)	< 0.001
Lost income due to pandemic	940 (22%)	351 (23%)	401 (18%)	409 (22%)	< 0.001
Actions					
Economic (borrowed money or used savings)	1428 (34%)	529 (34%)	837 (38%)	541 (29%)	< 0.001
Work (worked more, sought training)	279 (7%)	226 (15%)	154 (7%)	168 (9%)	< 0.001
Received gov support	332 (8%)	68 (4%)	221 (10%)	46 (2%)	< 0.001
Received family support	507 (12%)	114 (7%)	215 (10%)	98 (5%)

Respondents reported several actions taken in response to the climate hazards, combined into four categories. The most reported were the economic responses, such as borrowing money or using savings (n = 1428, 34% for floods; n = 529, 34% for drought; n = 837, 38% for hurricane; n = 541, 29% for heatwaves). Government support was most reported for those experiencing a hurricane (n = 221, 10%), or flooding (n = 332, 8%).

The logistic regression models highlight factors associated with each of the four types of response or action taken against a climate hazard. Compared to those who experienced flooding, respondents were more likely to take economic actions if they experienced a hurricane (OR = 1.19; 95% Confidence Interval 1.04, 1.35) (Table 3), and less likely to take economic action if they reported a heat wave (OR = 0.76; 95% CI 0.67, 0.86). Economic actions were more commonly reported by male respondents, and increased with age. Compared to respondents who were studying only, those who were working and studying were more likely to report economic action (OR = 1.52; 95% CI 1.37, 1.69). Compared to the poorest tercile, those in the middle or wealthiest tercile were less likely to employ an economic action. Lastly, those who lost income due to the pandemic were more likely to report an economic action due to climate (OR = 1.69; 95% CI 1.52, 1.87).

**Table 3:** Logistic regressions of factors associated with taking economic action (Model 1), work related actions (Model 2), receiving government support (Model 3) or receiving social network support (Model 4) in response to a climate event.

Variables	Model 1	Model 2	Model 3	Model 4
	Econ action taken	Work related action	Receive gov support	Receive social network support
Climate event: flood				
Drought	0.934 (0.814–1.071)	2.436*** (1.982–2.994)	0.462*** (0.342–0.624)	0.583*** (0.461–0.736)
Hurricane	1.185*** (1.044–1.345)	1.140 (0.901–1.441)	0.929 (0.754–1.144)	0.800** (0.657–0.974)
Heat	0.756*** (0.665–0.860)	1.412*** (1.141–1.749)	0.269*** (0.191–0.379)	0.402*** (0.315–0.512)
Gender (male)	REF	REF	REF	REF
Female	0.883*** (0.804–0.970)	1.049 (0.894–1.231)	0.987 (0.827–1.177)	1.374*** (1.176–1.606)
Trans/non-binary	0.733** (0.544–0.989)	1.345 (0.861–2.099)	0.762 (0.417–1.393)	1.075 (0.666–1.734)
18–24 years (vs 15–17 years)	1.251*** (1.103–1.419)	1.006 (0.814–1.244)	0.803 (0.615–1.049)	1.107 (0.904–1.355)
Working and Studying (Studying only)	REF	REF	REF	REF
Working only	1.593* (0.989–2.567)	1.681 (0.821–3.443)	1.941 (0.862–4.370)	0.880 (0.377–2.058)
Studying and working	1.523*** (1.373–1.690)	1.677*** (1.421–1.980)	0.961 (0.781–1.182)	0.829** (0.692–0.992)
Not studying and not working	0.978 (0.506–1.891)	1.120 (0.390–3.215)	1.563 (0.468–5.218)	0.449 (0.107–1.884)
Region: Central	REF	REF	REF	REF
South	1.020 (0.910–1.144)	0.874 (0.716–1.067)	1.943*** (1.597–2.364)	1.033 (0.861–1.239)
North-Central	1.042 (0.823–1.319)	0.902 (0.612–1.330)	0.319*** (0.140–0.724)	1.529** (1.089–2.146)
North-West	1.040 (0.788–1.373)	0.817 (0.495–1.348)	0.782 (0.428–1.430)	0.928 (0.586–1.468)
North	0.981 (0.832–1.157)	1.017 (0.779–1.329)	0.945 (0.670–1.334)	0.828 (0.628–1.091)
Rural (vs Urban)	1.016 (0.912–1.133)	1.093 (0.916–1.304)	1.519*** (1.252–1.842)	1.025 (0.859–1.223)
Wealth tertile (poorest Tertile)	REF	REF	REF	REF
Middle tertile	0.691*** (0.627–0.761)	0.927 (0.786–1.092)	1.342*** (1.117–1.614)	1.110 (0.950–1.296)
Wealthiest tertile	0.610*** (0.521–0.715)	0.872 (0.667–1.139)	1.398** (1.055–1.852)	0.993 (0.773–1.276)
Lost income due to pandemic	1.685*** (1.515–1.873)	1.312*** (1.103–1.562)	0.748** (0.596–0.939)	0.909 (0.759–1.088)
Constant	0.531*** (0.469–0.600)	0.057*** (0.046–0.071)	0.065*** (0.052–0.083)	0.111*** (0.091–0.135)
Observations	8729	8729	8729	8729

*Significant at P < 0.05, **P < 0.01, ***P < 0.001.

Respondents had an over two times higher odds of taking a work related action if the experienced drought (OR = 2.44, 95% CI 1.98, 2.99) or heat waves (OR = 1.41; 95% CI 1.14, 1.75) compared to floods (Table 3). Those who were studying and working were much more likely to employ a work related action (OR = 1.68; 95% CI 1.42, 1.98), and those who lost income due to the pandemic were also more likely to employ a work related action (OR = 1.31; 95% CI 1.10, 1.56). There were no significant differences by gender, age, region, or wealth tercile.

Receiving government support after a climate hazard was most reported by those who experienced  flooding or hurricanes, and was less common among those who experienced droughts (OR = 0.46; 95% CI 0.34, 0.62) or heatwaves (0.26; 95% CI 0.19, 0.38) (Table 3). Compared to the Central region of Mexico, those residing in the southern region had higher odds of receiving government support (OR = 1.94; 95% CI 1.60, 2.36) and those in Central-North had 68% lower odds of receiving government support (OR = 0.32; 95% CI 0.14, 0.72). Those in rural areas had higher odds of receiving government support (OR = 1.52; 95% CI 1.25, 1.84), as were those in the wealthiest tercile compared to the poorest tercile (OR = 1.40; 95% CI 1.06, 1.85). Those who lost income due to the pandemic were less likely to report receiving government support (OR = 0.75; 95% CI 0.60, 0.94).

Those who experienced flood events were the most likely to report relying on social networks and family for support. This was reported more by female respondents than male respondents (OR = 1.37; 95% CI 1.18, 1.61), and less likely to be reported for those who were working and studying vs studying only (OR = 0.83; 95% CI 0.69, 0.99). Relying on social networks and family for support was also more commonly reported among those living in Central North region compared to Central (OR = 1.52; 95% 1.09, 2.15). It was not associated with age, urban or rural, wealth tercile, or pandemic income loss.

**Figure 2:**
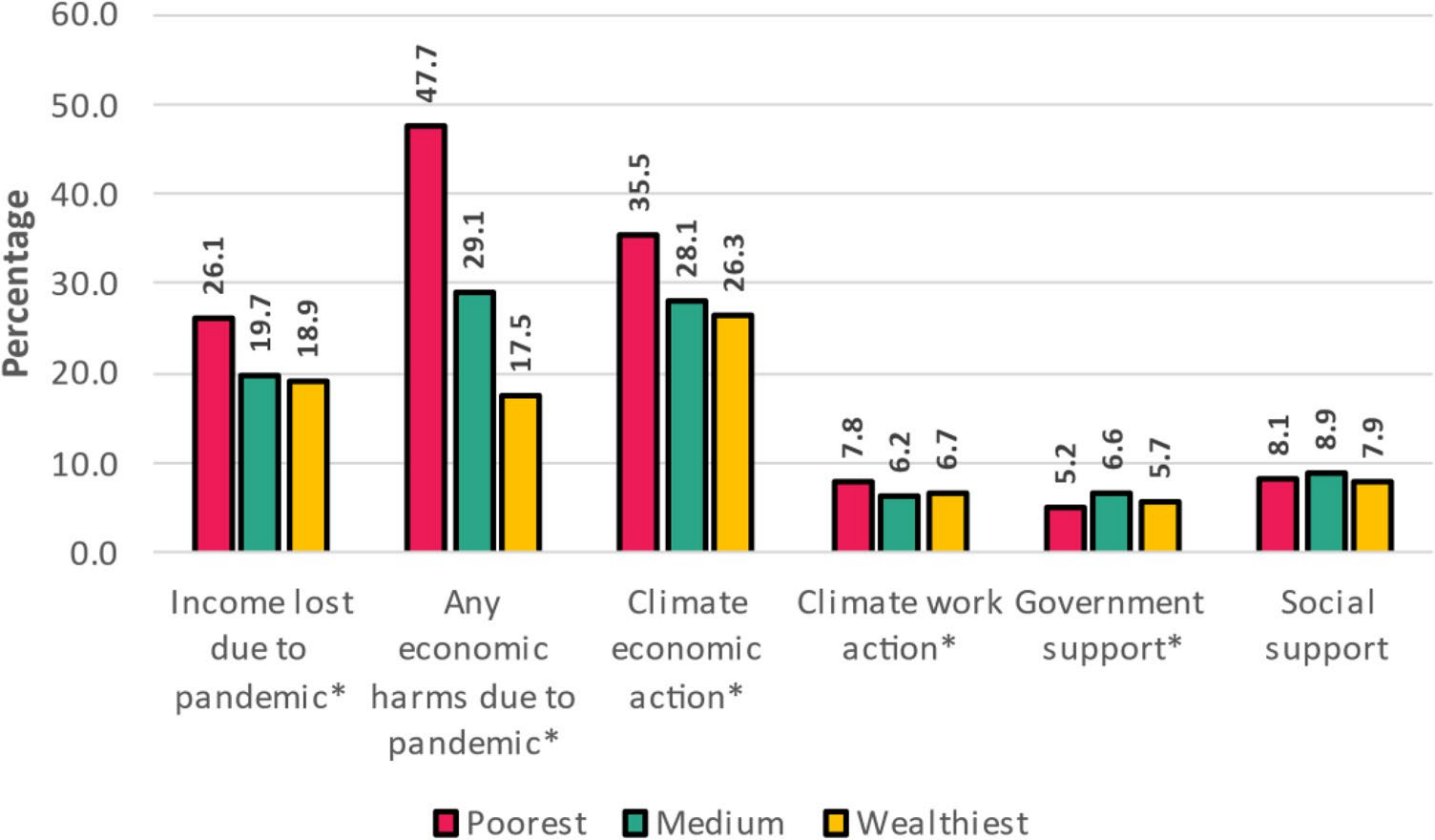
Proportion of adolescents and young adults that experienced pandemic related economic harm and the climate-related actions taken by wealth tertile. *Denotes statistical significance at P < 0.05.

Lastly, economic harms from both the pandemic and climate-related harms and actions were tabulated by wealth tercile. The poorest tercile was most likely to report income loss due to the pandemic, at least one economic harm due to the pandemic, and were most likely to report taking an economic action in response to a climate hazard (Fig. 2). Less than 10% reported a reliance on government support or social support in response to a climate hazard and the poorest wealth tercile were the least likely to report this.

## Discussion

Almost one in ten (8.1%) surveyed adolescents and young adults experienced a climate-related hazard in the last year, most commonly flooding events and hurricanes. Our findings show significant economic harms from the pandemic continuing into 2022 and overlapping with a range of economic adaptation strategies for climate hazards, showing aggravated economic losses. Our findings highlight geographical variation in exposure to climate events, with adolescents and young adults living in the Southern region most likely to report exposure to a recent climate event (mainly hurricanes). Those who were going to school and also working were more likely to report adverse economic impacts or work-related responses due to a climate event, suggesting they may be working earlier than planned to offset economic losses (due to climate and/or the pandemic) likely increasing risk of dropout. This rising generation of young people, particularly the most vulnerable, are being doubly impacted by climate and the pandemic, with long term-implications.

Our results highlight unequal exposure to and impacts of both climate hazards and COVID-19 harms, with the poorest individuals experiencing compounding harms. Respondents in the poorest tercile, mostly older boys, were most likely to report negative or maladaptive economic adaptation strategies in response to climate events, such as using savings or borrowing money. Adaptive capacity is closely linked to socioeconomic characteristics including economic assets, resources, wealth, and poverty^27^. The most vulnerable communities are those who are most exposed to hazardous climate events and have limited adaptive capacity^28^. Additionally, young people generally have fewer assets and live on lower incomes than older adults^23^. The economic impacts of climate events further constrain individuals’ ability to save money, reinforcing cycles of poverty. The pandemic also impacted households causing income loss and impeded their ability to buy food, purchase medicines, and pay important bills such as rent. These findings show how socioeconomic status limits individual adaptive capacity and amplifies the impact of climate events on the most vulnerable, highlighting the need for more macro-level interventions and government aid to protect young people and support long-term resilience in the wake of climate events.

Policies and government support must encompass differential vulnerability and capacity mechanisms in order for communities to effectively adapt to changing conditions and climate events^12^. Evidence shows inequalities in the distribution of civil protection resources used to respond to climate events, indicating that the most vulnerable geographic regions and individuals may not be appropriately targeted^29^. In this study, government support for climate events was most frequently reported among those who experienced hurricanes or flooding, and in rural areas, possibly because there are more emergency response mechanisms in place for acute events. Also clearly an event such as a hurricane may cause more infrastructure damage, and have more clear and immediate need for intervention, while an event like a heatwave may happen chronically and have less clearly linked policy implications. Flood events and hurricanes also tend to occur in Mexico’s most economically important States, likely directing government support and funds to these places first. It is also possible that the pandemic placed strains on resources and forced the government to shift aid priorities, resulting in low reporting of government assistance over the past year. More chronic events such as droughts may be receiving less government support and without structural changes will continue to cause economic and social harm to communities. Although our survey also did not differentiate the level of government support, it is important to note that there is a role for federal aid, but also value in local government responses for example early warning systems and support from state or municipal agencies.there is a role for federal aid there is also value in local government and response including early warning systems and support from state and municipal government agencies. Those in North-Central Mexico and mainly women were more likely to report social network support, potentially because this region is less likely to receive government support. Interestingly those economically harmed by the pandemic were less likely to report government support for climate, but this may relate to geography. Overall, COVID-19 may have shifted allocation of emergency government funds to the pandemic and away from climate over the pandemic period, though more research would be necessary to explore this.

Respondents who were exposed to a recent climate hazard were more likely to be in school and working as opposed to only in school, though only a small number of respondents reporting dropping out of school directly due to a recent climate event. Extreme weather events impact education by destroying school infrastructure, interrupting schedules, and draining finances away from educational services^30^. In addition to the impacts of climate events, recent studies show the COVID-19 pandemic caused a large decrease in time spent on school work (30% fewer hours) and an increase in the probability of working and in hours worked (though slightly mixed, potentially due to fewer jobs or work opportunities being available during the pandemic), particularly among boys^19^. Over the longer-term it will be important to understand how to mitigate these effects, either by facilitating young people’s return to school, or providing training and other opportunities. Rural communities that rely on agriculture may be particularly disrupted by climate change, as droughts harm agricultural livelihoods and shift young people’s work opportunities, in some settings driving them to work while studying or drop out entirely^23^. A previous study confirmed this pattern, finding households that experienced drought had lower per capita earnings, and were more likely to be poor after the drought, particularly if they are less familiar with relative water scarcity^25^.

This study has some limitations. VoCes is an online survey which presents opportunities, including reaching large numbers of young people, particularly during the peak of the pandemic when in-person data collection was halted. However, this format also presents several potential biases, including sampling and self-selection biases. Given the online nature of the survey, participation required access to digital resources and the survey therefore may not have reached the most vulnerable youth. Additionally, because respondents chose to participate, the extent to which their propensity for participating in the study was correlated with the topics of interest may produce bias. Relatedly, the survey said it was about COVID-19, which may have biased who took the survey. However, it did not mention climate change in the title or initial information. Finally, because this study used a convenience recruitment strategy that involved dissemination of the online survey through the Ministry of Education and Ministry of Health that are linked with (public) schools, a large proportion of respondents were students. This may have limited our inclusion of out-of-school youth, although additional outreach strategies included radio, tv, and other channels.

Mexico is one of the countries that has been most affected by the COVID-19 pandemic and is also particularly vulnerable to extreme climate events associated with climate and environmental change. To date, the majority of research investigating perceptions of climate change causes, impacts, and solutions has focused on adults^31^. Young people already do and will continue to disproportionately shoulder the impacts of both crises, exacerbating existing social and economic inequalities. Without direct investment in tackling the compounding harms of these dual crises disproportionately harming the poorest young people will drive inequality further. Engaging young people in climate responses and adaptation and ensuring equitable interventions and government aid that prioritizes protecting young people is crucial to long-term resilience in the wake of climate events.

## Conclusions

Our findings suggest that young people in Mexico are being harmed by exposure to climate events, with variation by demographic characteristics including gender and income. There is also variation in the types of harms experienced. For example, hurricanes or floods that are more acute events may cause more infrastructure damage and immediate impacts, whereas chronic events such as heatwaves may have less clear implications and may be increasingly becoming the norm. The pandemic highlights how other events can intersect with climate change to create worse economic and health outcomes. Future research should explore the most effective adaptive policies and responses, including considerations such as whether the support is federal or stems from more local intervention by states or municipalities. Additional exploration of how climate change harms more vulnerable groups is also needed. For example, how climate may shift time use and caregiving responsibilities in ways that exacerbate gender inequalities. This paper has mapped potential public policy gaps and opportunities to consider how to support vulnerable young people and vulnerable geographies across Mexico. As the pandemic and its effects wane, the climate crisis is accelerating, and our results highlight how vulnerabilities intersect. Further research to understand the dynamics between types of climate events, the degree of repeated exposure, individual and community behavioral responses, and the effectiveness of government policy mechanisms are critical.

## Data Availability

A cleaned, di-identified version of the dataset will be made publicly available for download via the Harvard Dataverse https://dataverse.harvard.edu/dataverse/popcouncil/?q=voces. The data created during the current study are available from the corresponding author and made available upon request.
